# miR-1-3p, as a novel diagnostic and prognostic biomarker, aggravates pancreatic acinar cell injury in acute pancreatitis by targeting GNPTAB

**DOI:** 10.1186/s41065-026-00704-8

**Published:** 2026-07-10

**Authors:** Haibin Huang, Jinyu Liu, Lianjun Wu, Wen Miao

**Affiliations:** 1 Department of Critical Care Medicine, The Third Affiliated Hospital of Guangzhou University of Chinese Medicine, Guangzhou, 510000 China; 2 Department of Emergency, Rudong People’s Hospital, Nantong, 226400 China; 3 Department of Surgery, The Third People’s Hospital of Cangnan County, Wenzhou, 325804 China; 4 Department of Emergency, The Affiliated Changzhou Hospital of Xuzhou Medical University, Changzhou Third People’s Hospital, No. 300, North Lanling Road, Tianning District, Changzhou City, Jiangsu Province 213000 China

**Keywords:** Acute pancreatitis, miR-1-3p, GNPTAB, Biomarker, Inflammation, Oxidative stress

## Abstract

**Objective:**

The mechanisms underlying the progression of acute pancreatitis (AP) remain incompletely elucidated. This study investigates the expression pattern, diagnostic and prognostic value of miR-1-3p in AP patients, and further elucidates its role in pancreatic acinar cell injury and the underlying molecular mechanisms.

**Methods:**

miR-1-3p levels were measured via qPCR and correlated with clinical indicators. ROC analysis evaluated diagnostic efficacy, and logistic regression assessed prognostic relevance. A Caerulein-induced injury model was established in MPC-83 cells. Post-transfection with a miR-1-3p inhibitor, cell viability (CCK-8), inflammatory factors (ELISA), and oxidative stress (WST-8/TBA) were evaluated. Bioinformatics-predicted target genes were validated through dual-luciferase reporter and RIP assays, with rescue experiments confirming the targeting relationship.

**Results:**

miR-1-3p was significantly upregulated in SAP patients and effectively distinguished healthy individuals (AUC = 0.880) and disease severity (AUC = 0.878). Its levels positively correlated with CRP, APACHE II, and BUN (*p* < 0.0001). High miR-1-3p expression independently predicted poor prognosis (OR = 9.54, *p* = 0.023). Inhibition of miR-1-3p alleviated Caerulein-induced cellular injury in vitro. GNPTAB was identified as a direct target, showing downregulation and negative correlation with miR-1-3p in patients. Rescue experiments confirmed that GNPTAB downregulation partially reversed the protective effects of miR-1-3p inhibition.

**Conclusions:**

miR-1-3p, upregulated in SAP, is a potential diagnostic biomarker and predictor of poor prognosis. It exacerbates pancreatic acinar cell injury by targeting GNPTAB, contributing to inflammation, oxidative stress, and AP progression.

**Supplementary Information:**

The online version contains supplementary material available at https://doi.org/10.1186/s41065-026-00704-8.

## Background

Acute pancreatitis (AP), a common gastrointestinal emergency [1], is characterized by a pathological process centered on localized pancreatic inflammation and acinar cell injury. This condition can rapidly progress to systemic inflammatory response syndrome and multiple organ dysfunction, posing a severe threat to patient life [2]. Although recent advances have been made in imaging diagnostics, the mechanisms underlying the progression of AP to severe forms remain incompletely understood. A major clinical challenge is the current lack of sensitive and specific biomarkers for early and accurate assessment of disease severity and prognosis. Therefore, identifying key regulatory molecules involved in the pathogenesis of AP and elucidating their mechanisms is of critical importance for improving clinical diagnosis and treatment strategies.

In recent years, microRNAs (miRNAs), a class of endogenous non-coding RNAs [3], have gained prominence for their critical role in regulating key pathophysiological processes such as inflammation, apoptosis, and autophagy through post-transcriptional gene regulation. As a result, they have become significant biomarkers and therapeutic targets across various diseases [4]. Within AP research, considerable evidence links the dysregulation of multiple miRNAs to both the onset and progression of the disease. One prominent example is serum hsa-miR-126-5p, which serves as a sensitive marker for distinguishing mild from severe AP [5]. Similarly, miR-325-3p, whose expression is notably downregulated in AP, mitigates pancreatic injury by targeting RIPK3 to inhibit necroptosis [6]. Additional miRNAs, including miR-216a, miR-216b, miR-217, miR-92b, miR-375, and miR-148a, have also been identified as potential serum biomarkers for AP [7]. Nevertheless, the precise role and mechanistic basis of miR-1-3p, a miRNA distinguished by its marked pancreatic tissue specificity and implicated as a potential key regulator in AP pathophysiology, remain incompletely defined. Several previous studies provide crucial clues regarding the role of miR-1-3p in AP. Bioinformatics analyses have predicted that hsa-miR-1-3p is a key molecule regulating core hub genes in AP, suggesting its potential central regulatory role in the pathogenesis of the disease [8]. Furthermore, miR-1-3p has been confirmed to exhibit significant pancreatic tissue specificity, and early studies identified it as one of the key markers for distinguishing pancreatic origin [9]. Additionally, miR-1-3p is a key regulator of metabolic homeostasis. Its circulating levels are elevated in prediabetes and show a positive correlation with insulin resistance [10, 11]. Given that metabolic dysfunction serves as both a significant trigger and a frequent pathological feature of AP, we hypothesize that miR-1-3p may function as a critical molecular link bridging metabolic stress and pancreatic inflammation.

Despite the compelling evidence highlighting the significance of miR-1-3p in AP, its clinical implications and biological functions remain insufficiently explored. Key aspects, including its expression profile in AP patients, diagnostic and prognostic potential, specific effects on acinar cell injury, and underlying molecular mechanisms, have yet to be elucidated. To address this gap, the present study systematically investigates the clinical relevance, biological functions, and mechanisms of miR-1-3p in acute pancreatitis. The findings may offer novel insights and potential strategies for early diagnosis, severity assessment, and targeted therapy in AP.

## Methods

### Study population

In this study, 123 consecutive patients with AP were retrospectively enrolled from the Emergency Department and Gastroenterology Department between January 2022 and December 2023. Based on the Atlanta Classification criteria [12], patients were stratified into non-severe (Non-SAP, *n* = 73) and severe (SAP, *n* = 50) groups. All AP diagnoses conformed to the Chinese guidelines for acute pancreatitis management [13]. Exclusion criteria included coexisting pancreatic diseases, malignancies, significant organ dysfunction, and pregnancy. Additionally, 93 age- and sex-matched healthy individuals from routine health screenings served as controls. All AP patients underwent one-year telephone follow-up for outcome assessment. The Ethics Committee of Rudong People’s Hospital approved this study. The study was conducted in accordance with the Declaration of Helsinki. Written informed consent was obtained from all participants.

### Cell culture and treatment

The murine pancreatic acinar cell line MPC-83 was acquired from the ATCC. Cells were cultured in DMEM with 10% FBS, 100 U/mL penicillin, and 100 µg/mL streptomycin at 37℃ in a 5% CO_2_ humidified incubator. MPC-83 cells in their logarithmic growth phase were seeded into 6-well plates at a density of 1 × 10⁵ cells per well. Once cells reached 70% to 80% confluence, the culture medium was replaced with medium containing 10 nM Caerulein, and cells were incubated for 12 h to establish an in vitro acute pancreatitis injury model. Control cells received an equivalent volume of phosphate-buffered saline.

### Cell transfection and grouping

Four experimental groups were designed to assess miR-1-3p function: Control, Caerulein, Caerulein+inhibitor-NC, and Caerulein + miR-1-3p inhibitor. At 70–80% confluence, cells were transfected following Lipofectamine™ 3000 instructions. Briefly, 5 µL transfection reagent and 100 pmol miRNA inhibitor were each diluted in 125 µL Opti-MEM, incubated for 20 min at room temperature, and mixed to form transfection complexes. Complexes were added to cells cultured in antibiotic-free medium with 2% FBS. After 6 h, medium was changed to complete culture medium. Twenty-four hours after transfection, all groups except Control were treated with 10 nM Caerulein for 12 h, while the Control group received equal-volume PBS. Cells and supernatants were collected for further assays.

### qRT-PCR

Quantitative real-time PCR analysis was conducted to examine the expression levels of target molecules. For miRNA detection, reverse transcription was performed with a stem-loop RT kit specifically designed for miRNA analysis, supplied by TaKaRa, Japan. mRNA was reverse transcribed using PrimeScript RT Master Mix from the same manufacturer. The resulting cDNA was amplified with SYBR Green Premix Pro Taq HS qPCR Kit, produced by Accurate Biology, China. Amplification and fluorescence detection were carried out on a QuantStudio 5 real-time PCR instrument from Applied Biosystems, USA. U6 snRNA and GAPDH were utilized as internal reference controls for normalizing miR-1-3p and GNPTAB expression, respectively. Relative quantification of gene expression was calculated through the 2^−ΔΔCt^ method.

### CCK‑8 assay

Cell viability was assessed using a CCK-8 kit (Dojindo, Japan). After transfection and Caerulein treatment, cells from each group were seeded into 96-well plates at 5 × 10³ cells per well and incubated at 37 °C with 5% CO₂. Viability was measured at 0, 24, 48, and 72 h by adding 10 µL of CCK-8 solution to each well, incubating for 2 h, and reading the absorbance at 450 nm with a BioTek (USA) microplate reader.

### Detection of inflammatory factors and oxidative stress

Culture supernatants were collected, centrifuged to remove debris, and assayed for inflammatory cytokines using ELISA kits from Wuhan Huamei Biological Engineering Co., Ltd. (China) according to the manufacturer’s instructions. Absorbance at 450 nm was read on a BioTek (USA) microplate reader, and concentrations were determined against standard curves. Oxidative stress was evaluated by measuring SOD activity with the WST-8 method (Beyotime) and MDA content via the TBA method using reagents from Nanjing Jiancheng Bioengineering Institute.

### Bioinformatics analysis and mechanistic validation

Potential target genes of miR-1-3p were predicted using the TargetScan, miRWalk and miRDB databases. The screening criteria were set as follows: TargetScan Total context + + score ≤ -0.11, miRDB Target Score ≥ 70, and miRWalk binding free energy ≤ -17 kcal/mol. The intersection of the predictions from the three databases yielded a list of 36 common candidate target genes. Among these, GNPTAB was prioritized for further validation based on its established functional relevance to pancreatic pathology, including its role in lysosomal dysfunction, acinar cell regeneration, and cholesterol metabolism in pancreatitis [14, 15]. Based on these predictions, sequences containing either the wild-type WT or mutant MUT binding site within the GNPTAB gene 3 prime UTR were synthesized and cloned into the pGL3 reporter vector from Promega. The resulting reporter plasmids were then co-transfected into MPC-83 cells along with either the miR-1-3p mimic or its negative control mimic NC. After 48 h, luciferase activity was measured using the Dual-Luciferase Reporter Assay System also from Promega, and the values were normalized against Renilla luciferase activity.

To verify direct binding, an RNA immunoprecipitation RIP experiment was performed. Cell lysates from transfected MPC-83 cells were subjected to immunoprecipitation using the EZ-Magna RIP Kit from Millipore with an anti-Ago2 antibody or a control IgG antibody. Finally, qRT-PCR was employed to assess the enrichment of GNPTAB mRNA within the immunoprecipitated complexes.

### Statistical analysis

Statistical analyses were conducted with SPSS 26.0 and GraphPad Prism 9.0. Normally distributed continuous variables are expressed as mean ± standard deviation and compared using the independent samples t‑test or one‑way ANOVA with LSD post‑hoc test. Categorical variables are presented as counts and analyzed by the chi‑square test. The diagnostic value of miR‑1‑3p was evaluated via ROC curve analysis, and its association with prognosis was examined by multivariate logistic regression. The multivariate logistic regression model included the following covariates: APACHE II score, Ranson’s score, CRP, BUN, ALB, and miR-1-3p. Multicollinearity among the covariates in the multivariate logistic regression model was assessed using the VIF, with VIF values < 5 indicating no significant collinearity. A *p*‑value < 0.05 was considered statistically significant.

## Results

### Baseline clinical characteristics of the study population

A total of 123 AP patients and 93 healthy controls were included. Comparisons between the AP group and the healthy controls revealed no significant differences in age, gender, BMI, prevalence of diabetes mellitus, or hypertension (Table 1, all *p* > 0.05). However, significant differences were observed between the two groups in the levels of CRP, BUN, and ALB (Table 1, all *p* < 0.001). Furthermore, among the AP patients, stratification by severity into Non-SAP (*n* = 73) and SAP (*n* = 50) groups was performed. As summarized in Table 2, no significant differences existed between the two groups regarding age, gender, BMI, diabetes, or hypertension history (*p* > 0.05). In contrast, the SAP group exhibited significantly higher APACHE II scores, Ranson scores, serum C‑reactive protein, and blood urea nitrogen, along with notably lower serum albumin levels compared to the Non-SAP group (all *p* < 0.001).

**Table 1:** Baseline analysis between healthy control and AP groups

Variable	Healthy control (*n* = 93)	AP (*n* = 123)	*p*-value
Age (year)	51.87 ± 4.51	51.39 ± 6.08	0.505
Gender (male/female)	48/45	64/59	0.952
BMI (kg/m^2^)	24.01 ± 1.23	24.36 ± 1.76	0.091
Diabetes mellitus (yes/no)	29/64	34/89	0.573
Hypertension (yes/no)	27/66	37/86	0.868
CRP (mg/L)	7.06 ± 1.07	99.51 ± 66.92	< 0.001
BUN (mmol/L)	3.59 ± 0.66	5.80 ± 1.63	< 0.001
ALB (g/L)	44.12 ± 3.17	35.71 ± 5.52	< 0.001

*BMI* body mass index, *CRP* C-reactive protein, *BUN* blood urea nitrogen, *ALB* albumin

**Table 2:** Baseline analysis between Non-SAP and SAP groups

Variable	Non-SAP (*n* = 73)	SAP (*n* = 50)	*p*-value
Age (year)	51.37 ± 5.72	51.42 ± 6.62	0.964
Gender (male/female)	38/35	26/24	0.995
BMI (kg/m^2^)	24.18 ± 1.95	24.61 ± 1.42	0.182
Diabetes mellitus (yes/no)	18/55	16/34	0.375
Hypertension (yes/no)	24/49	13/37	0.418
APACHE II score	7.53 ± 2.89	15.72 ± 4.00	< 0.001
Ranson’s score	1.88 ± 0.74	5.00 ± 1.05	< 0.001
CRP (mg/L)	47.22 ± 8.19	175.86 ± 32.08	< 0.001
BUN (mmol/L)	4.87 ± 0.99	7.17 ± 1.39	< 0.001
ALB (g/L)	38.18 ± 4.60	32.11 ± 4.72	< 0.001

*BMI* body mass index, *APACHE* acute physiology and chronic health evaluation, *CRP* C-reactive protein, *BUN* blood urea nitrogen, *ALB* albumin

### The expression pattern of miR-1-3p and its diagnostic value for AP

**Fig. 1:**
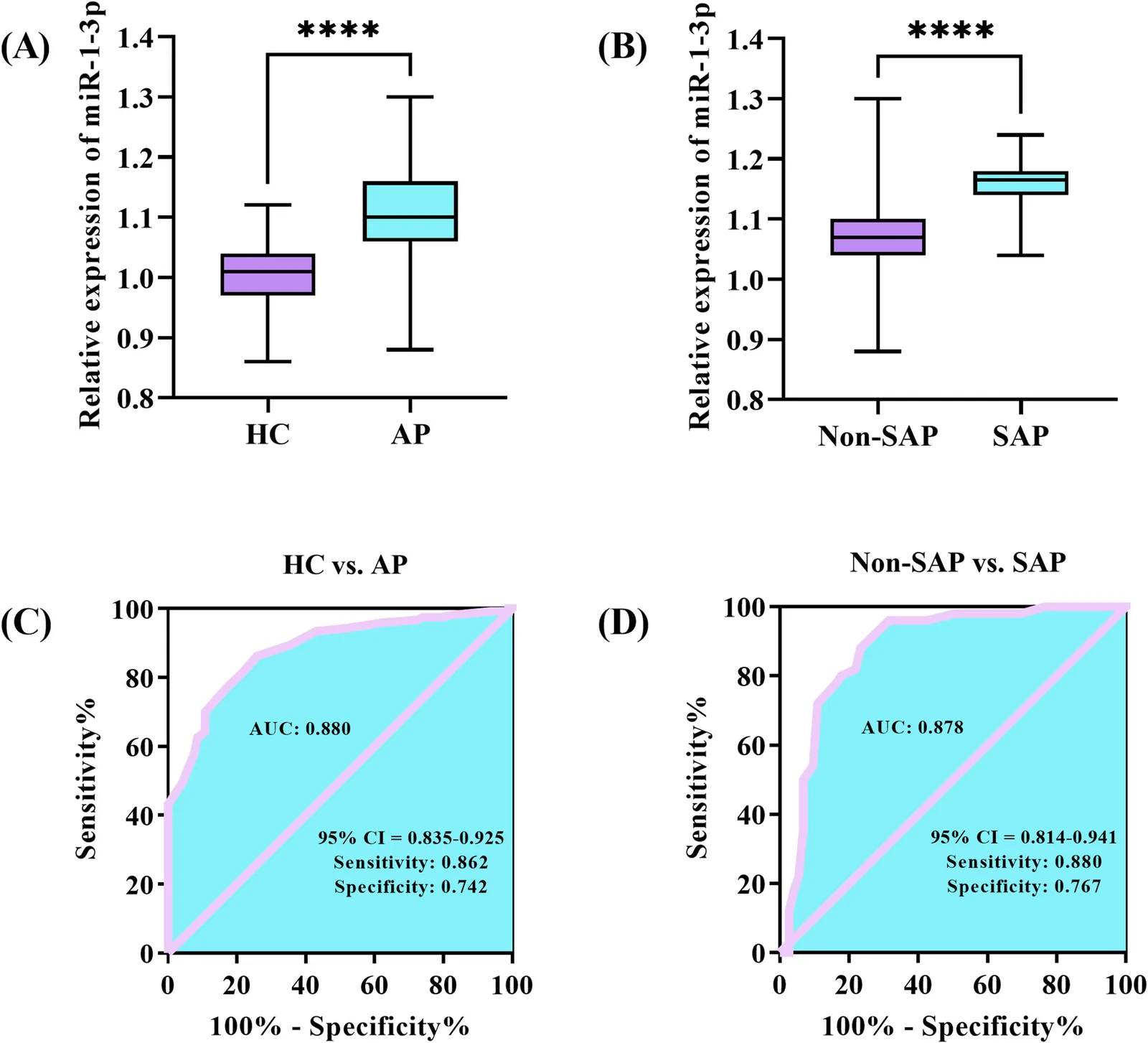
Expression of miR-1-3p in patients with acute pancreatitis (AP) and its diagnostic value. **(A)** Relative expression of miR-1‑3p in AP patients versus healthy controls. **(B)** Relative expression of miR‑1‑3p in Non‑SAP versus SAP patients. **(C)** ROC curve of miR‑1‑3p for diagnosing AP. **(D)** ROC curve of miR‑1‑3p for distinguishing Non‑SAP from SAP. ****P < 0.0001

Compared with the healthy control HC group, acute pancreatitis AP patients exhibited a marked upregulation in miR‑1‑3p expression (Fig. 1A, *p* < 0.001). Notably, miR‑1‑3p levels were significantly higher in patients with severe acute pancreatitis SAP relative to those in the non‑severe acute pancreatitis Non‑SAP group (Fig. 1B, *p* < 0.001). To further evaluate the discriminative potential of miR‑1‑3p, receiver operating characteristic ROC curve analyses were performed. miR‑1‑3p demonstrated effective differentiation between healthy controls and AP patients, with an area under the curve AUC of 0.880 (95% confidence interval CI: 0.835–0.925). The sensitivity and specificity for this distinction reached 86.2% and 74.2% respectively (Fig. 1C). Furthermore, miR‑1‑3p displayed excellent diagnostic performance in distinguishing Non‑SAP from SAP patients, yielding an AUC of 0.878 (95% CI: 0.814–0.941) with a sensitivity of 88.0% and a specificity of 76.7% (Fig. 1D).

### Correlation between miR-1-3p expression and clinical severity indicators

**Fig. 2:**
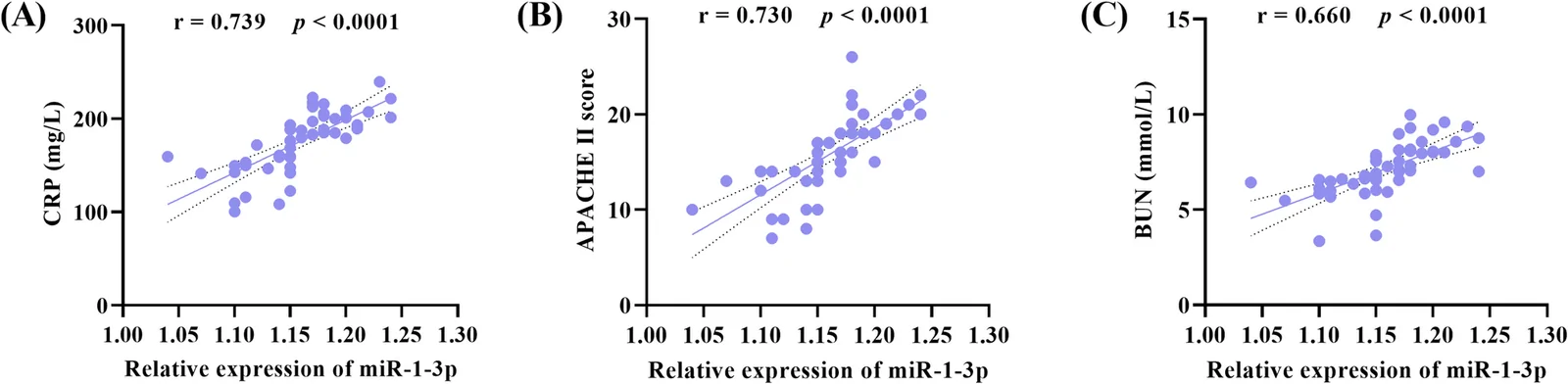
Correlation between miR‑1‑3p expression and clinical parameters in AP patients. Correlation of miR‑1‑3p with **(A)** CRP, **(B)** APACHE II score, and **(C)** BUN

To explore the relationship between miR-1-3p levels and disease severity, correlation analyses were conducted with key clinical parameters. The relative level of miR-1-3p demonstrated a strong positive correlation with serum C-reactive protein concentration (Fig. 2A, *r* = 0.739). A similarly significant positive correlation was observed with APACHE II scores (Fig. 2B, *r* = 0.730). Furthermore, miR-1-3p expression also showed a notable positive correlation with blood urea nitrogen levels (Fig. 2C, *r* = 0.660). All reported correlations were statistically significant with *p*-values less than 0.0001.

### Predictive value of miR‑1‑3p for clinical outcomes in SAP patients

After a one-year follow-up of 50 SAP patients, two groups were defined according to clinical outcomes: favorable prognosis (*n* = 34) and unfavorable prognosis (*n* = 16). Baseline characteristics such as age and gender were comparable between groups (Table 3). However, patients with unfavorable outcomes had significantly higher APACHE II scores, Ranson scores, CRP, and BUN levels, along with lower albumin levels (*p* < 0.05). Importantly, serum miR‑1‑3p expression was markedly higher in the unfavorable prognosis group (Table 3, *p* < 0.0001). Univariate logistic regression showed a significant association between elevated miR‑1‑3p and adverse outcomes (Table 4). Multivariate logistic regression analysis was performed with adjustments for APACHE II score, Ranson’s score, CRP, BUN, ALB, and miR-1-3p. After adjusting for other clinical parameters in multivariate analysis, high miR‑1‑3p expression (OR = 9.54, 95% CI: 1.36–66.90, *p* = 0.023) and elevated BUN (OR = 7.22, 95% CI: 1.06–48.97, *p* = 0.043) remained independent risk factors for poor prognosis. The multivariate logistic regression model demonstrated excellent discrimination with a C-statistic of 0.918 (95% CI: 0.845–0.991, *p* < 0.0001) (Supplementary Fig. 1). The Hosmer-Lemeshow test yielded a p-value of 0.659, indicating good calibration. Multicollinearity assessment showed that all VIF values were below 5, indicating no significant collinearity among the covariates (Supplementary Table 1).

**Table 3:** Baseline characteristics of patients with SAP, stratified by clinical outcome

Variable	clinical outcome		*p*-value
	Favorable (*n* = 34)	Unfavorable (*n* = 16)	
Age (year)	51.35 ± 6.21	51.56 ± 7.64	0.918
Gender (male/female)	18/16	9/7	0.831
BMI (kg/m^2^)	24.45 ± 1.44	24.97 ± 1.34	0.232
Diabetes mellitus (yes/no)	20/14	11/5	0.510
Hypertension (yes/no)	19/15	9/7	0.981
APACHE II score	14.88 ± 3.71	17.50 ± 4.13	0.029
Ranson’s score	4.68 ± 0.88	5.69 ± 1.08	0.001
CRP (mg/L)	166.71 ± 31.22	195.3 ± 25.00	0.002
BUN (mmol/L)	6.78 ± 1.29	8.00 ± 1.27	0.003
ALB (g/L)	33.15 ± 4.16	29.89 ± 5.19	0.021
miR‑1‑3p	1.14 ± 0.04	1.19 ± 0.03	< 0.001

*BMI* body mass index, *APACHE* acute physiology and chronic health evaluation, *CRP* C-reactive protein, *BUN* blood urea nitrogen, *ALB* albumin

**Table 4:** Univariate and multivariate logistic regression analysis of factors associated with unfavorable clinical outcomes in patients with SAP

Variables	Univariate OR (95% CI)	*p*-value	Multivariate OR (95% CI)	*p*-value
miR-1-3p	6.19 (1.48–25.85)	0.012	9.54(1.36–66.90)	0.023
Age (year)	1.63 (0.49–5.39)	0.425	—	—
Gender	1.14 (0.35–3.78)	0.827	—	—
BMI (kg/m^2^)	2.79 (0.79–9.78)	0.110	—	—
Diabetes mellitus	1.54 (0.44–5.42)	0.501	—	—
Hypertension	1.02 (0.31–3.36)	0.981	—	—
APACHE II score	3.55 (1.01–12.57)	0.049	3.39(0.51–22.49)	0.206
Ranson’s score	4.875 (1.17–20.26)	0.029	8.48(0.96–74.54)	0.054
CRP (mg/L)	4.33 (1.04-18.00)	0.044	8.54(0.93–78.93)	0.059
BUN (mmol/L)	4.03 (1.13–14.36)	0.031	7.22(1.06–48.97)	0.043
ALB (g/L)	0.233 (0.062–0.875)	0.031	0.20(0.03–1.44)	0.110

Odds ratios (OR) and 95% confidence intervals (CI) are presented

*BMI* body mass index, *APACHE* acute physiology and chronic health evaluation, *CRP* C-reactive protein, *BUN* blood urea nitrogen, *ALB* albumin

### Role of miR‑1‑3p in caerulein-induced murine pancreatic acinar cell injury model

**Fig. 3:**
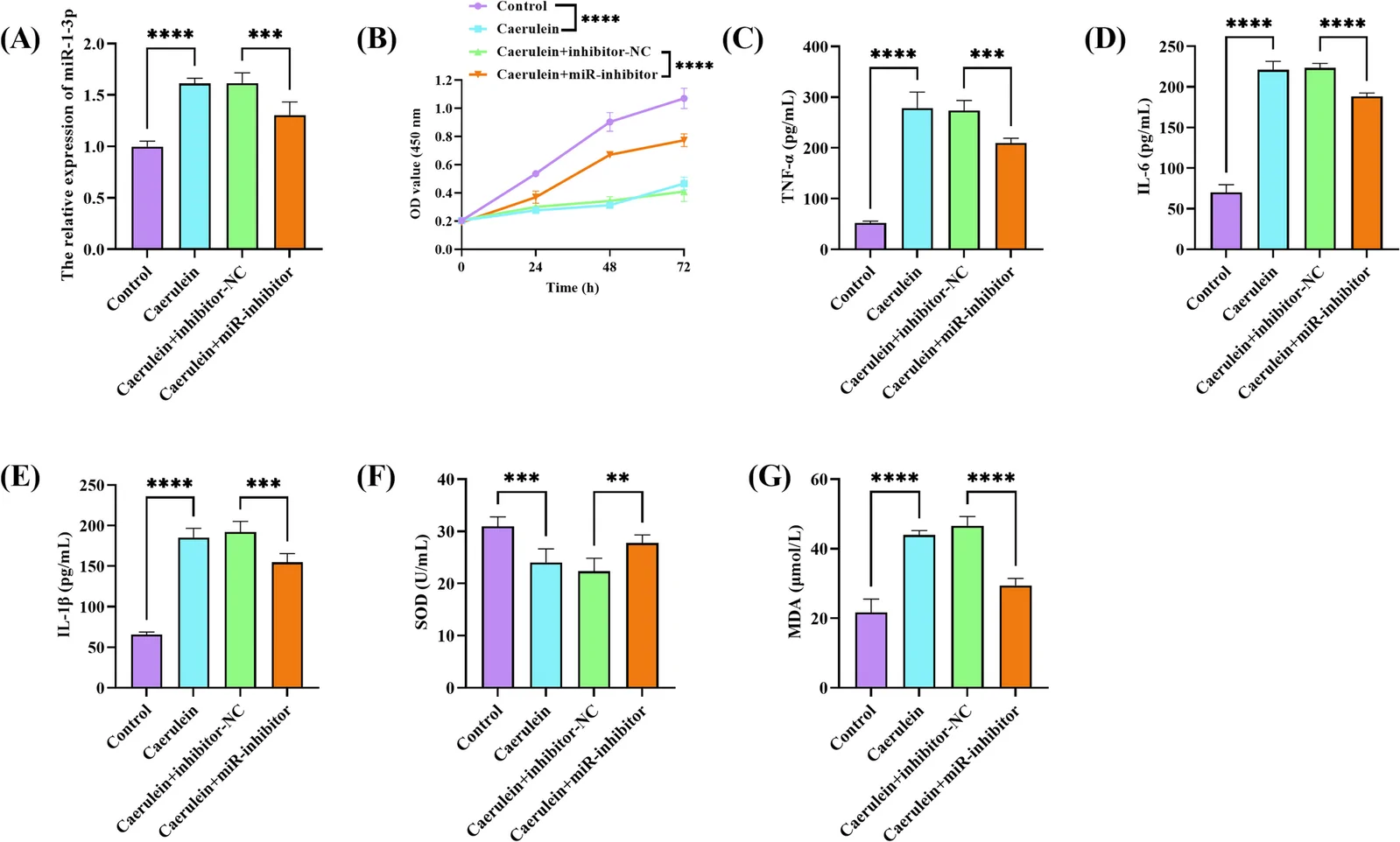
Protective effect of miR‑1‑3p inhibition on Caerulein‑induced pancreatic cell injury. **A** Relative expression of miR‑1‑3p in the Control, Caerulein, Caerulein+inhibitor‑NC, and Caerulein + miR‑1‑3p inhibitor groups. **B** Changes in cell viability among the groups. Expression levels of inflammatory cytokines: **C** TNF‑α, **D** IL‑6, **E** IL‑1β. Oxidative stress indicators: **F** SOD, **G** MDA. ****p < 0.0001, ***p < 0.001, **p < 0.01

To investigate the specific function of miR‑1‑3p in acute pancreatitis in vitro, a cell injury model was established by treating murine pancreatic acinar MPC‑83 cells with Caerulein. Results indicated that Caerulein treatment significantly increased miR‑1‑3p expression in MPC‑83 cells (*p* < 0.0001). Transfection with an miR‑1‑3p inhibitor effectively reversed this upregulation (Fig. 3A, *p* < 0.001). Furthermore, Caerulein stimulation markedly reduced cell viability, while co‑transfection with the miR‑1‑3p inhibitor significantly restored cell survival (Fig. 3B, *p* < 0.0001). Additional analyses revealed that Caerulein treatment led to a pronounced elevation in the release of pro‑inflammatory cytokines, including TNF‑α (Fig. 3C), IL‑6 (Fig. 3D), and IL‑1β (Fig. 3E), all with *p* < 0.0001. Concurrently, SOD activity was notably decreased (Fig. 3F, *p* < 0.001), while MDA content was increased (Fig. 3G, *p* < 0.0001). It is worth noting that inhibition of miR‑1‑3p expression substantially reversed these pathological alterations, effectively lowering the levels of inflammatory cytokines and improving cellular antioxidant capacity.

### Validation of the relationship between GNPTAB and miR‑1‑3p

**Fig. 4:**
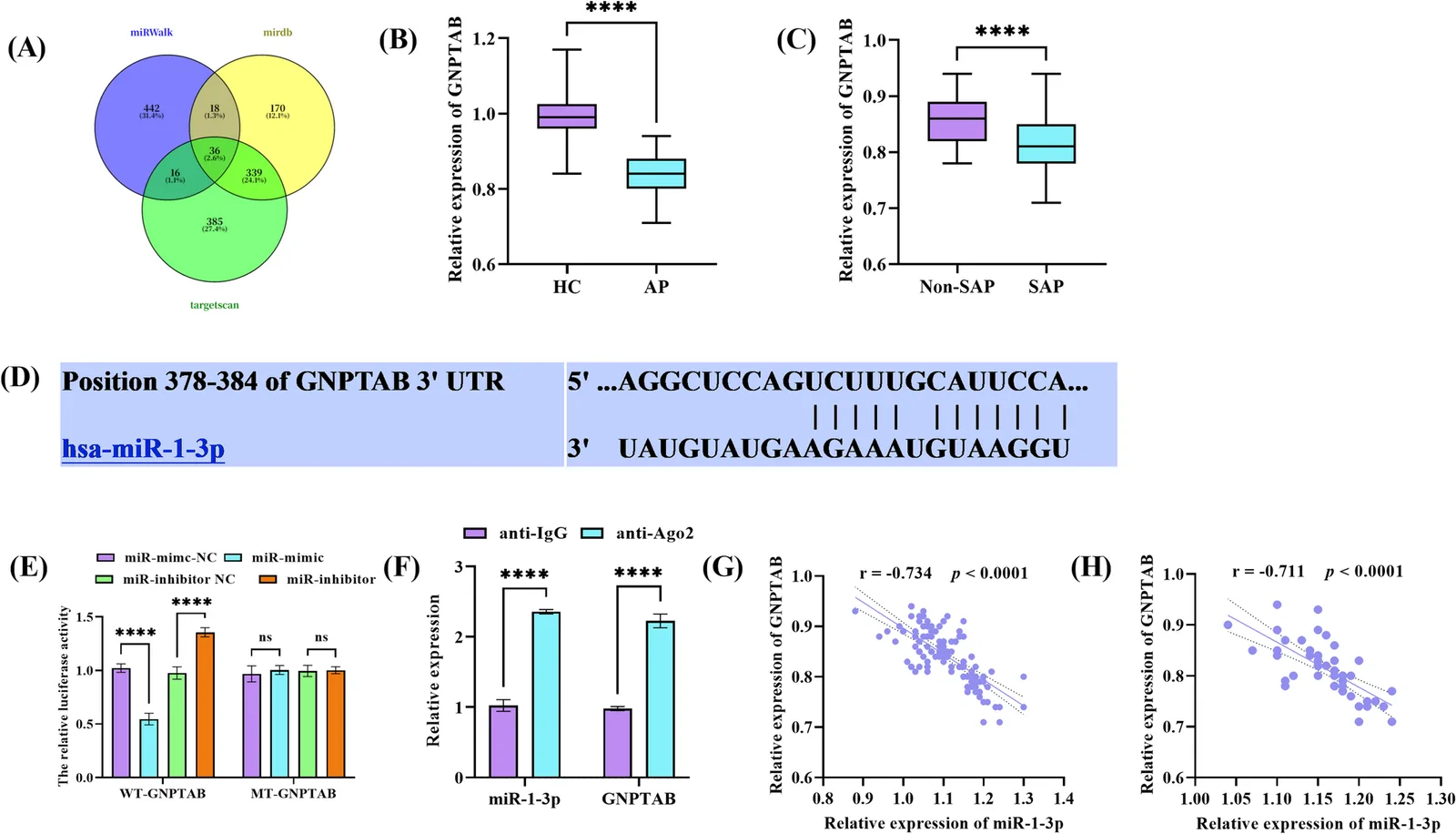
Mechanistic validation of the interaction between miR‑1‑3p and GNPTAB. **A** Venn diagram showing the overlap of target genes predicted by miRWalk, miRDB, and TargetScan for miR-1-3p. **B** Expression of GNPTAB in AP patients and healthy controls. **C** Expression of GNPTAB in Non‑SAP versus SAP patients. **D** Binding sequence of miR‑1‑3p to the GNPTAB 3’‑UTR. **E** Dual‑luciferase reporter assay confirming the binding of miR‑1‑3p to GNPTAB. **F** RIP assay showing enrichment of miR‑1‑3p and GNPTAB in the Ago2 complex. **G** Negative correlation between miR‑1‑3p and GNPTAB expression in AP patients. **H** Negative correlation between miR‑1‑3p and GNPTAB expression in SAP patients. ****p < 0.0001

The intersection of predictions from TargetScan, miRDB, and miRWalk databases yielded 36 common candidate target genes (Fig. 4A). Among these, GNPTAB was selected for further validation. The predicted binding between miR-1-3p and GNPTAB was collectively supported by a seed match, a 14 bp binding region, a miRDB Target Score of 77, a TargetScan Total context + + score of -0.14, and a binding free energy of -18.2 kcal/mol. In patient samples, GNPTAB mRNA levels were significantly lower in AP patients than in healthy controls (Fig. 4B, *p* < 0.0001) and further decreased in SAP compared with Non‑SAP patients (Fig. 4C, *p* < 0.0001). Sequence alignment showed complementarity between the miR‑1‑3p seed region and nucleotides 378–384 in the GNPTAB 3’‑UTR (Fig. 4D). Overexpression of miR‑1‑3p mimic notably reduced the luciferase activity of the wild‑type GNPTAB reporter but not the mutant reporter (Fig. 4E, *p* < 0.0001). RIP assays confirmed enrichment of both miR‑1‑3p and GNPTAB mRNA with anti‑Ago2 antibody compared to anti‑IgG (Fig. 4F, *p* < 0.0001). A significant negative correlation was observed between serum miR‑1‑3p and GNPTAB mRNA in AP patients (Fig. 4G, r=‑0.734, *p* < 0.0001) and in the SAP subgroup (Fig. 4H, r=‑0.711, *p* < 0.0001).

### GNPTAB mediates the regulatory effects of miR-1-3p on cellular injury

**Fig. 5:**
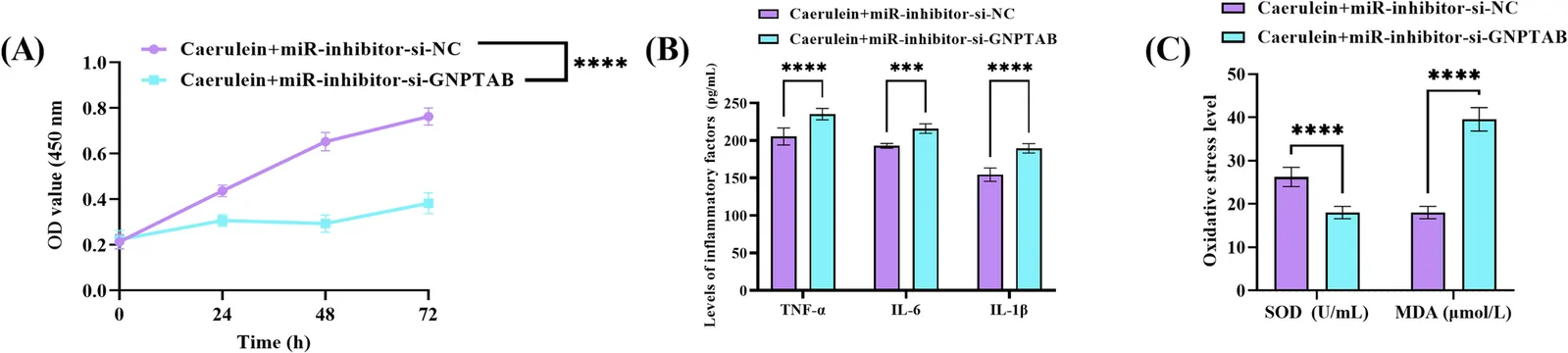
Knockdown of GNPTAB partially reverses the protective effect of the miR‑1‑3p inhibitor. **A** Knockdown of GNPTAB reversed the effect of the miR‑1‑3p inhibitor on cell viability. **B** Knockdown of GNPTAB reversed the miR‑1‑3p inhibitor‑mediated suppression of inflammatory cytokines (TNF‑α, IL‑6, and IL‑1β). **C** Knockdown of GNPTAB also reversed the effects of the miR‑1‑3p inhibitor on oxidative stress markers, as determined by SOD activity and MDA levels. ****p < 0.0001, ***p < 0.001

To determine whether GNPTAB mediates the functional effects of miR‑1‑3p, rescue experiments were conducted. Knocking down GNPTAB expression concurrently with the inhibition of miR‑1‑3p partially reversed the protective effect of the miR‑1‑3p inhibitor on cell viability (Fig. 5A, *p* < 0.0001). Further analyses indicated that the anti‑inflammatory and antioxidant effects produced by miR‑1‑3p inhibition were correspondingly attenuated when GNPTAB expression was suppressed. This was reflected by increased levels of TNF‑α, IL‑6, and IL‑1β in the cell culture supernatant (Fig. 5B, *p* < 0.0001). Concurrently, SOD activity decreased while MDA content increased (Fig. 5C, *p* < 0.0001).

## Discussion

Acute pancreatitis is a rapidly progressive clinical emergency with variable outcomes, where severe cases frequently present systemic inflammatory response and multiple organ dysfunction, resulting in high mortality [16]. Compared with conventional biomarkers, miRNAs exhibit significant clinical potential due to their stability, tissue specificity, and regulatory role in pathological processes [17]. With the maturation and wider accessibility of high‑throughput sequencing and qPCR technologies, miRNA detection costs have substantially declined. This economic improvement enables the feasible application of miRNA profiling for large‑scale clinical screening and dynamic monitoring of disease progression.

miRNAs are highly stable in serum/plasma, and a single baseline measurement can reliably reflect expression levels at admission, as demonstrated by numerous miRNA biomarker studies using single sampling [18–20]. Previous investigations have associated miR‑1‑3p with metabolic disturbances and pancreatic inflammation. However, its precise functional mechanism in acute pancreatitis remains insufficiently defined. This molecule also exhibits dysregulated expression in pathological states such as pancreatic ductal adenocarcinoma, where it correlates closely with malignant progression [21, 22], suggesting that miR‑1‑3p may function as a shared regulatory element across the spectrum of pancreatic disorders. The present study demonstrates for the first time that serum levels of miR‑1‑3p are significantly elevated in patients with AP and increase progressively with disease severity, effectively distinguishing among healthy controls, Non‑SAP, and SAP individuals. This finding aligns with a prior report describing altered serum miR‑1‑3p expression in SAP patients [23], thereby confirming the expression profile of this molecule in AP. In clinical AP assessment, CRP is commonly used for early evaluation of inflammatory severity [24], while the APACHE II score provides comprehensive disease assessment and prognostic prediction [25]. Elevated blood urea nitrogen BUN reflects intravascular volume depletion, and the integration of these three parameters facilitates precise AP stratification [26]. In this study, miR‑1‑3p showed significant positive correlations with CRP, APACHE II score, and BUN, indicating that beyond reflecting local pancreatic inflammation, it may also participate in regulating systemic inflammatory responses and functional impairment. Additionally, given that dysregulation of miR-1-3p has also been reported in pancreatic ductal adenocarcinoma [21, 22] and prediabetes [11], this molecule may not be entirely specific to acute pancreatitis. Regarding the comparison between miR-1-3p and conventional clinical markers, it should be noted that in the present study cohort, conventional markers (CRP, APACHE II, Ranson score, BUN, and ALB) showed extremely significant differences between groups with minimal overlap in value distributions. Under such circumstances, direct ROC comparison may not accurately reflect the relative diagnostic performance of these markers and could be misleading. Therefore, we did not perform such analysis. However, the value of miR-1-3p lies not in replacing existing markers but in providing complementary information. Our work focused specifically on patients with acute pancreatitis. We provide further evidence that miR-1-3p acts as an independent predictor of poor outcomes in this population. Its ability to distinguish disease severity also highlights its potential as a novel diagnostic biomarker. Together, these findings offer new perspectives on the pathogenesis of acute pancreatitis and provide experimental grounds for developing diagnostic and therapeutic strategies centered on miR-1-3p.

Caerulein, a cholecystokinin‑like peptide, is widely used to establish in vitro acute pancreatitis models because it mimics key pathological processes of AP by persistently activating the zymogen secretion pathway in pancreatic acinar cells [27]. Previous studies have demonstrated that various miRNAs are involved in the regulatory network of Caerulein‑induced AP through distinct mechanisms. For instance, miR‑339‑3p expression is downregulated in Caerulein‑treated cells, and it alleviates inflammation and apoptosis by targeting TRAF3 [28]. Similarly, miR‑605‑3p expression decreases after Caerulein stimulation and attenuates ductal cell injury via modulating the DUOX2/NLRP3 pathway [29]. In the present study, miR‑1‑3p expression was significantly upregulated upon Caerulein stimulation and correlated with reduced cell viability, increased inflammatory cytokine release, and elevated oxidative stress. Application of an miR‑1‑3p inhibitor effectively reversed these injury‑related changes, indicating that miR‑1‑3p promotes cellular damage in this model, likely by exacerbating inflammation and oxidative stress.

Mechanistically, we demonstrate that miR‑1‑3p aggravates pancreatic acinar cell injury by targeting GNPTAB, which encodes the lysosomal enzyme‑sorting enzyme N‑acetylglucosamine‑1‑phosphotransferase. GNPTAB dysfunction is linked to lysosomal storage disorders [30], and its downregulation in pancreatitis impairs acinar cell regeneration [15] and promotes lysosomal dysfunction and cholesterol metabolic disturbance [14]. In AP patients, GNPTAB expression was decreased and negatively correlated with miR‑1‑3p levels. Direct binding between miR‑1‑3p and the GNPTAB 3ʹ‑UTR was confirmed by dual‑luciferase and RIP assays. Inhibiting miR‑1‑3p alleviated Caerulein‑induced cellular injury, inflammation, and oxidative stress, effects partially reversed by GNPTAB knockdown. These findings suggest that miR‑1‑3p exacerbates AP by repressing GNPTAB, disrupting lysosomal function, and amplifying inflammatory and oxidative damage, thereby linking organelle dysfunction to AP pathogenesis.

However, our understanding of miR-1-3p and its target GNPTAB primarily relies on an in vitro Caerulein-induced cellular model, which does not fully replicate the complex pathophysiology of human acute pancreatitis. To address this limitation, we plan to conduct in vivo experiments using a Caerulein-induced mouse model with miR-1-3p antagomir injection to validate the miR-1-3p/GNPTAB axis under physiological conditions. Second, among the 36 common candidate target genes identified, this study focused on GNPTAB due to scope and resource constraints, and the remaining genes warrant future investigation. Third, serum miR-1-3p levels were measured only at admission, and longitudinal data on dynamic changes are lacking, which limits our ability to define its optimal clinical use case. Fourth, the absence of disease control cohorts (e.g., pancreatic cancer, rheumatoid arthritis) limits the assessment of miR-1-3p specificity for AP. Fifth, the current findings are derived from a single-center retrospective cohort, which may limit their generalizability, and larger prospective or external validation studies are needed. To address this, we have initiated plans to collect external samples in collaboration with two nearby hospitals, with an estimated addition of 80–100 AP patients. Despite these limitations, our findings provide a solid foundation for future research. Addressing these issues in subsequent studies will help clarify the clinical utility of miR-1-3p and accelerate its potential translation into diagnostic and therapeutic applications for acute pancreatitis.

## Conclusions

This study demonstrates that miR‑1‑3p is upregulated in severe acute pancreatitis SAP, indicating its potential as both a diagnostic and prognostic biomarker. In Caerulein‑induced AP cell models, increased miR‑1‑3p expression promotes the release of inflammatory cytokines, enhances oxidative stress, and reduces cell viability, whereas inhibiting miR‑1‑3p effectively reverses these pathological changes. Moreover, miR‑1‑3p directly binds and represses GNPTAB expression, thereby exacerbating cellular damage. These findings not only deepen the understanding of AP pathogenesis, but also provide a new direction for developing therapeutic strategies targeting the miR‑1‑3p/GNPTAB axis. 

## Supplementary Information

Supplementary Material 1: Figure 1. ROC curve of the multivariate logistic regression model for predicting unfavorable prognosis in SAP patients.

Supplementary Material 2.

## Data Availability

The datasets used and/or analysed during the current study are available from the corresponding author on reasonable request.
