# Supplementary material for: miR-1-3p, as a novel diagnostic and prognostic biomarker, aggravates pancreatic acinar cell injury in acute pancreatitis by targeting GNPTAB
Source: Hereditas. 2026 Jul 10;163:103. doi: 10.1186/s41065-026-00704-8 (PMC13640367; doi:10.1186/s41065-026-00704-8)
Supplement: Supplementary file 1 [file 41065_2026_704_MOESM2_ESM.docx]

Supplementary Table 1 Multicollinearity assessment of variables included in the multivariate logistic regression model

| Variable | VIF |
| --- | --- |
| miR-1-3p | 1.16 |
| APACHE II score | 1.20 |
| Ranson’s score | 1.04 |
| CRP (mg/L) | 1.09 |
| BUN (mmol/L) | 1.01 |
| ALB (g/L) | 1.14 |
